# Anchoring Pd Single Atoms on N‐Doped MOF‐Derived TiO_2_ for Photocatalytic Hydrogen Evolution With an Ultra‐High Rate up to 185.8 mmol g^−1^ h^−1^

**DOI:** 10.1002/advs.78590

**Published:** 2026-11-09

**Authors:** Xingyan Yang, Guangri Jia, Wenhao Li, Mingzhu Xie, Yong Shuai, Zhaolong Wang

**Affiliations:** ^1^ School of Energy Science and Engineering Harbin Institute of Technology Harbin People's Republic of China; ^2^ Zhengzhou Advanced Research Institute of Harbin Institute of Technology Zhengzhou People's Republic of China

**Keywords:** N doping, Pd single‐atom, Photocatalytic H_2_ evolution, TiO_2_

## Abstract

Photocatalytic hydrogen (H_2_) evolution provides a sustainable pathway for clean energy production. However, conventional photocatalysts suffer from wide bandgaps and rapid charge recombination, leading to low photocatalytic efficiency. Herein, we construct an asymmetric N─Pd─O coordination environment by anchoring Pd single atoms (SAs) onto N‑doped MIL‑125‑derived TiO_2_. The optimized 1%PdN‑TiO_2_ catalyst achieves a high H_2_ evolution rate up to 185.8 mmol g^−1^ h^−1^ and an apparent quantum efficiency of 67.4% both measured under 365 nm monochromatic light irradiation. Synchrotron X‑ray absorption fine structure spectroscopy, ultrafast spectroscopy, and density functional theory calculations reveal that the asymmetric N─Pd─O coordination promotes directional charge transfer and lowers the H_2_ evolution reaction (HER) barrier. This work offers insights into the rational design of heteroatom‑modulated asymmetric coordination microenvironments toward high‑efficiency MOF‑derived single‑atom photocatalysts for sacrificial‑agent‑assisted photocatalytic H_2_ production.

## Graphical abstract

N doping is adopted to introduce abundant binding sites within TiO_2_ matrix, which enables firm immobilization of isolated Pd single atoms. The as‐constructed PdN‐TiO_2_ single atom photocatalyst achieves greatly promoted solar‐driven hydrogen evolution performance.

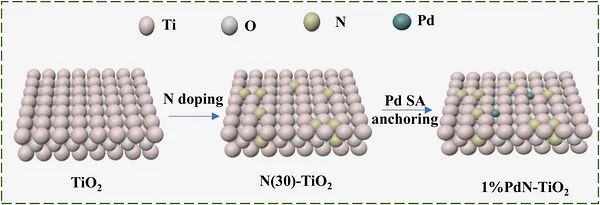

## 1 Introduction

The global energy crisis and environmental degradation urgently demand the development of clean and renewable energy sources [1, 2, 3]. Photocatalytic water splitting for H_2_ production has emerged as a promising pathway to directly convert solar energy into chemical fuels, wherein the development of high performance photocatalysts is key to enhance light utilization and charge separation efficiency [4]. Herein, H_2_ production is realized in a water‑methanol mixture, where methanol serves as an irreversible sacrificial electron donor to consume photogenerated holes. Highperformance photocatalysts are critical to improve light utilization and charge‑separation efficiency for this half‑reaction process. Metal‐organic frameworks (MOFs), featuring tunable topologies, high surface areas, and abundant active sites, have attracted significant attention in photocatalysis [5, 6, 7]. However, the wide bandgap of most MOFs limits their visible light response and photocatalytic activity. Among various modification strategies, nonmetal doping and noble metal loading have been validated to be the most effective methods [8]. Tao et al. [9] demonstrated that N doping introduces oxygen vacancies (O_v_) in TiO_2_, narrowing the bandgap and achieving a 5.5‐fold enhancement in HER.

Beyond nonmetal doping, anchoring noble metal single‐atom (SA) cocatalysts onto TiO_2_ represents another powerful strategy to capture photogenerated electrons and suppress recombination [10, 11]. SAs offer maximized atom utilization and unique electronic structures [12, 13, 14]. However, conventional SA catalysts typically present symmetric M─O─Ti coordination environments, which often hinder directional charge transfer. Recent studies have sought to overcome such a limitation. Xiao et al. [15] revealed that N doping modulates d‐p‐band electronic coupling between Cu SAs and N‐doped TiO_2_, inducing electron redistribution that optimizes HER kinetics. Zhao et al. [16] showed that Pd SAs synergize with O_v_ on TiO_2_ to reduce the Gibbs free energy of H_2_ adsorption. Despite these advances, deliberately constructing an asymmetric N─Pd─O coordination environment within a MOF‐derived TiO_2_ framework remains underexplored.

Herein, we anchor Pd SA atoms onto N‐doped MIL‐125‐derived TiO_2_ to construct an asymmetric N─Pd─O coordination environment. Experimental and theoretical analyses reveal that N doping not only narrows the bandgap and extends visible‐light absorption but also stabilizes Pd SAs through Pd‐N bonds, thereby enhancing metal–support interactions. The optimized 1% PdN‐TiO_2_ catalyst achieves a high H_2_ evolution rate up to 185.8 mmol g^−1^ h^−1^ with an apparent quantum efficiency (AQE) of 67.4% under 365 nm irradiation. Synchrotron XAFS, ultrafast spectroscopy, and DFT calculations further demonstrate that the asymmetric N─Pd─O coordination promotes directional charge transfer and lowers the reaction barrier. This work establishes heteroatom‐induced asymmetric coordination as a viable design principle for MOF‐derived single‐atom photocatalysts.

## 2 Results and Discussion

### 2.1 Catalysts Characterizations

The PdN‐TiO_2_ photocatalyst was synthesized through the multi‐step reactions outlined in Figure 1a. X‐ray diffraction (XRD) presents that MIL‐125 precursor matches well with literature patterns [17], confirming successful MOF synthesis (Figure S1). The well‐crystallized MOF precursor serves as an ideal template for subsequent N doping and Pd anchoring after calcination, as it provides abundant unsaturated Ti sites and a porous framework. The pristine TiO_2_ exhibits a typical anatase phase (Figure 1b). Importantly, all modified samples N (30)‐TiO_2_, 1% Pd‐TiO_2_, and 1% PdN‐TiO_2_ retain the same anatase structure without detectable peak shifts, indicating that neither N doping nor Pd SA anchoring disrupts TiO_2_ lattice framework. No characteristic peaks for N or Pd‐related phases are observed (Figure S2), suggesting the high dispersion of both dopants. Inductively coupled plasma optical emission spectrometry (ICP‐OES) results (Figure S3) further confirm that the actual Pd loading (0.96 wt.%) closely matches the theoretical value (1.0 wt.%), demonstrating good compositional controllability. The slight deviation is likely within the experimental error range of the ICP‐OES measurement.

**FIGURE 1:**
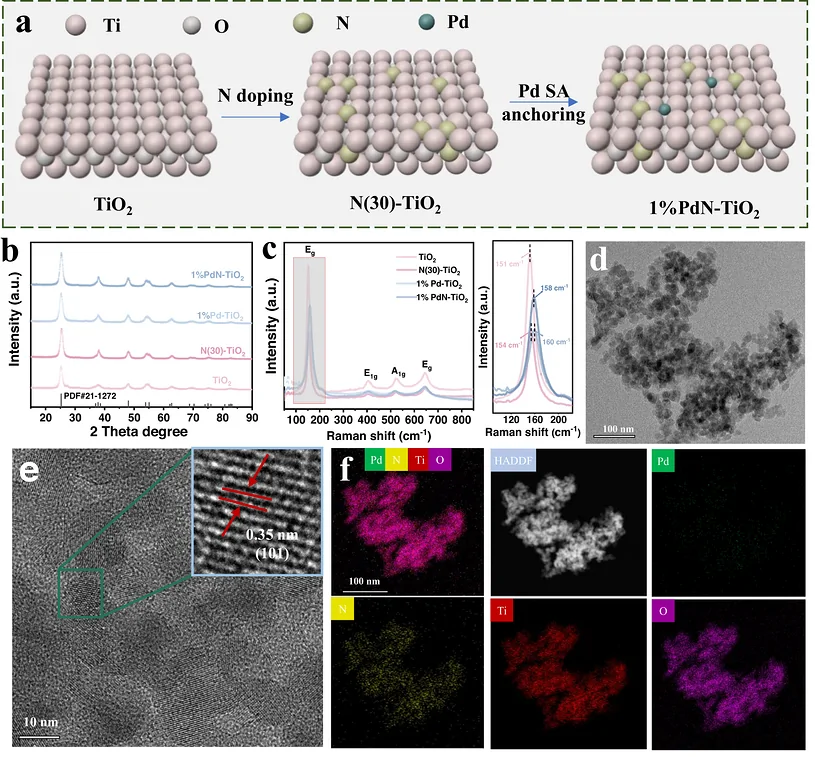
Synthesis and structure of the photocatalysts. (a) Synthetic process of photocatalysts. (b) XRD patterns and (c) Raman spectra of TiO_2_, N (30)‐TiO_2_, 1% Pd‐TiO_2_, and 1% PdN‐TiO_2_. (d, e) TEM images and (f) STEM‐EDS mapping of 1%PdN‐TiO_2_.

Raman spectroscopy was employed to probe local lattice distortions induced by N doping (Figure 1c). The pristine TiO_2_ shows characteristic anatase peaks at approximately 151 cm^−1^ (E_g_), 399 cm^−1^ (B_1g_), 513 cm^−1^ (A_1g_ + B_1g_), and 639 cm^−1^ (E_g_) [18]. For N‐containing samples N (30)‐TiO_2_ and 1% PdN‐TiO_2_, these peaks broaden and shift to higher wavenumbers, particularly 151 cm^−1^ peak which splits into two components at 154 and 158 cm^−1^. Such behavior is consistent with the incorporation of N into TiO_2_ lattice or surface interstitial sites. This incorporation creates local distortion and defect sites, which may act as anchoring sites for foreign metal atoms. Thus, N doping modifies the TiO_2_ lattice and generates defect sites with the potential to immobilize Pd SAs. The Fourier Transform Infrared Spectroscopy (FTIR) spectra of all samples exhibit O─H (3400 cm^−1^) and H─O─H (1630 cm^−1^) bands from adsorbed water/hydroxyls (Figure S4). All samples retain the characteristic TiO_2_ peaks, indicating an intact main framework. Distinct extra absorption signals in the range of 1000‐1500 cm^−1^ are observed for N(30)‑TiO_2_ and 1% PdN‑TiO_2_, evidencing successful N doping. These bands originate from Ti‑N stretching and interstitial N‑H bending vibrations, whereas Ti─O lattice vibrations mainly occur below 1000 cm^−1^. For 1% PdN‑TiO_2_, the altered band intensity within this window further suggests Pd‑N coordination between Pd SA and doped N. In contrast, 1% Pd‑TiO_2_ presents no extra signals in this region, indicating that Pd is loaded as metal without forming new chemical bonds.

The specific surface area and pore structure of the samples were investigated by N_2_ adsorption‐desorption measurements at 77 K. The specific surface area follows an increasing order: TiO_2_< N (30)‐TiO_2_< 1%Pd‐TiO_2_< 1% PdN‐TiO_2_ (Figure S5). Notably, 1% PdN‐TiO_2_ delivers the largest specific surface area of 190.7 m^2^ g^−1^. A higher specific surface area generally provides more exposed catalytic centers and active sites, which benefits the regulation and stabilization of Pd coordination environments. Furthermore, the synergistic contribution of enlarged surface area and suitable porosity enables 1% PdN‐TiO_2_ to achieve more efficient mass transfer and boosted photocatalytic performance. The pore volumes of TiO_2_, N (30)‐TiO_2_, 1% Pd‐TiO_2_, and 1% PdN‐TiO_2_ are respectively determined to be 0.13, 0.43, 0.50, and 0.56 mL g^−1^ (Figure S6).

Correspondingly, 1% PdN‐TiO_2_ shows the most abundant mesoporous structures among all of those SAs, as reflected by the shape and hysteresis of the isotherm curves. The transmission electron microscopy (TEM) image (Figure 1d) reveals that 1% PdN‐TiO_2_ sample is composed of nanoscale grains. Moreover, the lattice fringes with a spacing of approximately 0.35 nm can be observed (Figure 1e), which corresponds to the interplanar spacing of **anatase**‐phase TiO_2_ [19]. Energy‐dispersive X‐ray spectroscopy (EDS) elemental mapping was performed to characterize the distribution of Pd, N, Ti, and O. Superimposed maps confirm uniform co‐localization of all elements [20]. Pd, N, Ti, and O are homogeneously dispersed (Figure 1f).

The transient photocurrent response curve reveals that 1% PdN‐TiO_2_ exhibits the highest and most stable photocurrent density under periodic illumination (Figure 2a), with a peak current substantially higher than those of pristine TiO_2_, N (30)‐TiO_2_, and 1%Pd‐TiO_2_. The results demonstrate that the synergistic coupling between N doping and Pd SAs efficiently promotes photogenerated carrier separation and accelerates interfacial charge transfer kinetics [21]. Electrochemical impedance spectroscopy further elucidates the inherent charge transport behavior. EIS was measured at 0.198 VRHE with a frequency range of 10^5^–0.1 Hz and an amplitude of 5 mV. Quantitative equivalent‑circuit fitting for Nyquist plots was implemented, and the fitted charge‑transfer resistance (R_ct_) values are summarized in Table S1. Generally, a smaller semicircle diameter in the Nyquist plot reflects lower interfacial charge transfer resistance [22]. The semicircle diameters follow the sequence: TiO_2_ > N (30)‐TiO_2_ > 1% Pd‐TiO_2_> 1% PdN‐TiO_2_ (Figure 2b). The fitted R_ct_ follows the identical trend: TiO_2_ (161530 Ω) > N(30)‑TiO_2_ (73950 Ω) >1% Pd‑TiO_2_ (40210 Ω) >1% PdN‑TiO_2_ (12040 Ω). Such a trend confirms that the co‐modulation of N doping and Pd SAs constructs a highly efficient electron conduction pathway, thereby effectively inhibiting the recombination of photogenerated electron‐hole pairs. Figure 2c shows the linear sweep voltammetry (LSV) curves of different catalysts. Quantitatively, at a fixed current density of −0.5 mA cm^−2^, 1% PdN‑TiO_2_ requires the most positive potential of −0.64 V vs NHE. These results confirm that 1% PdN‑TiO_2_ exhibits the smallest overpotential for HER, indicating its remarkable reducing capability [23]. This interpretation is corroborated by both the high photocurrent density (Figure 2a) and the minimized Rct obtained from EIS fitting (Figure 2b), which collectively demonstrate fast interfacial charge‐transfer kinetics.

**FIGURE 2:**
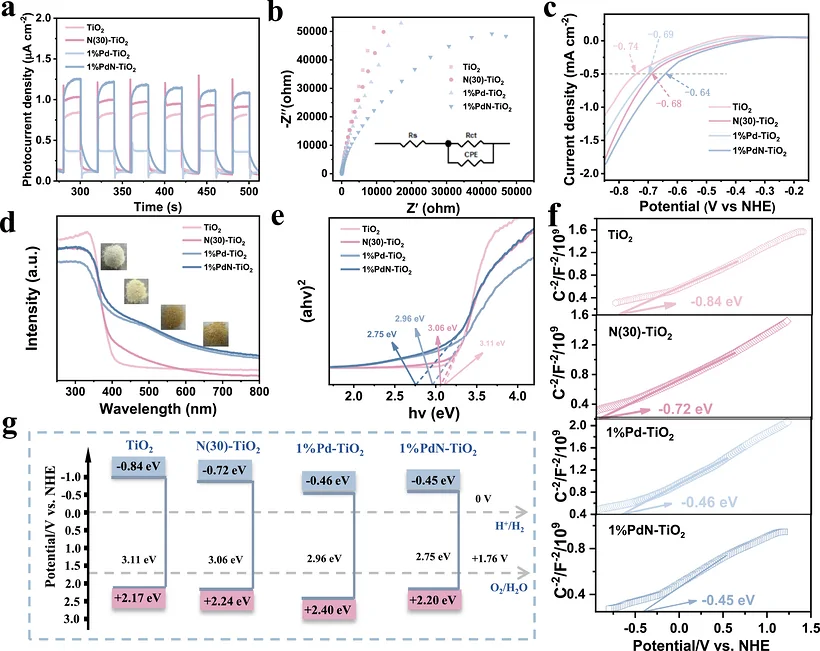
Photoelectrochemical and optical properties of the photocatalysts. (a) Transient photocurrent response, (b) EIS plots, and (c) Electrochemical linear sweep voltammetry of TiO_2_, N (30)‐TiO_2_, 1% Pd‐TiO_2_, and 1% PdN‐TiO_2_. (d) UV–vis spectra and (e) Tauc diagrams converted from UV–vis of TiO_2_, N (30)‐TiO_2_, 1% Pd‐TiO_2_, and 1% PdN‐TiO_2_. (f) Mott–Schottky curves and g energy band structure diagram.

UV–vis diffuse reflectance spectroscopy was employed to investigate the influence of atomic coordination modulation on the optical absorption behavior of the catalysts (Figure 2d) [24]. Pristine TiO_2_ exhibits strong light absorption only in the ultraviolet region below 380 nm, while N (30)‐TiO_2_ displays an evidently broadened light‐response range. Compared with other samples, 1% PdN‐TiO_2_ presents further enhanced visible‐light absorption intensity, accompanied by a remarkable red shift of the absorption edge. Based on the Kubelka–Munk equation [25] and Tauc plot calculations (Figure 2e), pristine TiO_2_ exhibits a wide bandgap of 3.11 eV, while N (30)‐TiO_2_, 1% Pd‐TiO_2_, and 1% PdN‐TiO_2_ display gradually narrowed bandgaps of 3.06, 2.96, and 2.75 eV, respectively. Mott–Schottky (M–S) measurements were further performed to evaluate the flat‐band potential and interfacial carrier concentration of the photocatalysts (Figure 2f). The M ‐S test was performed at a fixed frequency of 1000 Hz, with an AC amplitude of 10 mV. The applied DC potential scanning range was set from −1 to +1 V versus open‑circuit potential and 5 cycles were collected for each sample. All samples present positive slopes in the M–S plots, demonstrating the typical electronic characteristics of n‐type semiconductors. The flat‑band potentials and quantitatively calculated donor carrier density (N_d_) for each sample are compiled in Table S2. The detailed Mott‑Schottky formula used for Nd calculation is provided in Supplementary Information. The corresponding flat‐band potentials (vs. Ag/AgCl) are determined to be −0.84, −0.72, −0.46, and −0.45 eV, respectively.

According to semiconductor energy band theory, the conduction band potential of n‐type semiconductors is approximately 0.1 V more positive than the flat‐band potential versus the normal H_2_ electrode (NHE). Accordingly, the conduction band positions of TiO_2_, N (30)‐TiO_2_, 1% Pd‐TiO_2_, and 1% PdN‐TiO_2_ are systematically calculated. Combined with the formula *E_g_* = *E_VB_* − *E_CB_*, the valence band potentials can be derived from the measured bandgap and conduction band potentials [26, 27]. Compared with pristine TiO_2_ (−0.84 eV vs. NHE), 1% PdN‐TiO_2_ delivers a remarkable positive shift of the flat‐band potential to −0.45 eV versus NHE, accompanied by the highest N_d_ among all samples. This positive shift places the conduction band edge closer to the H^+^/H_2_ reduction potential (0 V vs. NHE), thereby lowering the thermodynamic overpotential for proton reduction and facilitating the HER. These results confirm that the synergistic integration of N doping and Pd SA engineering effectively modulates the electronic band structure, strengthens the photoreduction capability of photogenerated electrons, and accelerates interfacial charge reaction kinetics. Combined with the bandgap and flat‐band potential data, a comprehensive energy band alignment diagram is finally established (Figure 2g).

### 2.2 Photocatalytic H_2_ Evolution Performance

The photocatalytic H_2_ evolution performance of the as‐prepared catalysts was evaluated under a 365 nm LED irradiation source using a multi‐channel photochemical reaction system. According to the optimization results in Figure S7, deionized (DI) water/methanol mixed solution (volume ratio 1:2) was selected as the reaction medium. This ratio provides adequate methanol to scavenge photogenerated holes without deteriorating catalyst dispersion. Isotope‐labeling experiments, in which water and methanol were replaced with equal volumes of D_2_O and CD_3_OD, respectively, confirm that water is the dominant H_2_ source for photocatalytic H_2_ production (see Figure S8 for detailed discussion). The reaction temperature is stabilized at 40°C by circulating cooling water to avoid solvent evaporation at elevated temperature, which would alter solution composition during long‑term irradiation (Figure S9). To clarify the contribution of N doping to photocatalytic activity, H_2_ evolution rates of a series of N‐gradient doped samples are systematically compared. The N (30)‐TiO_2_ delivers a moderate H_2_ evolution rate of 5.83 mmol g^−1^ h^−1^, preliminarily verifying that N doping effectively optimizes the optical absorption behavior and enhances light utilization efficiency of pristine TiO_2_ (Figure 3a). The highest H_2_ evolution rate is achieved at 30 mg CO(NH_2_)_2_, suggesting an optimal N doping level that balances bandgap narrowing, lower doping levels insufficiently extend light absorption, while excessive doping may introduce recombination centers that offset the benefits.

**FIGURE 3:**
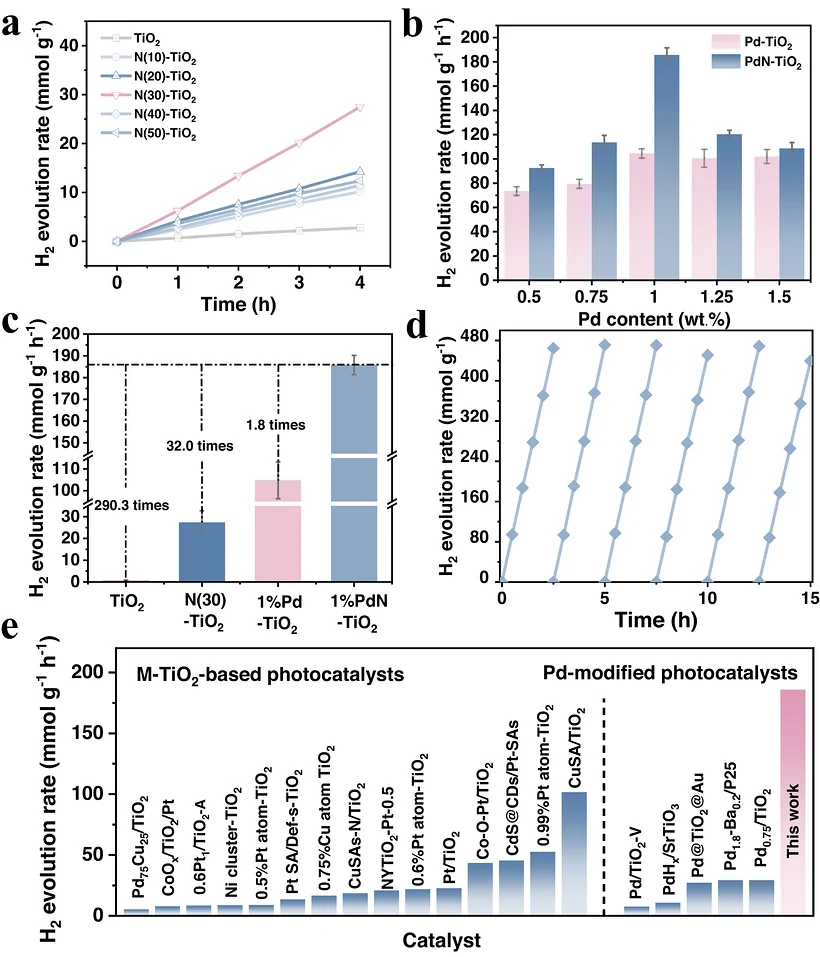
PHE activity evaluation of the photocatalysts. (a) H_2_ evolution rate of N(*x*)‐TiO_2_ (*x* = 10, 20, 30, 40, 50). (b) Photocatalytic H_2_ evolution rates of Pd‐TiO_2_ and PdN‐TiO_2_ with different Pd contents. (c) Photocatalytic H_2_ evolution rates of TiO_2_, N (30)‐TiO_2_, 1%Pd‐TiO_2_, and 1%PdN‐TiO_2_. (d) Photocatalytic H_2_ evolution cycling stability of 1%PdN‐TiO_2_. (e) Photocatalytic H_2_ evolution summary of the previously reported M‐TiO_2_‐based (M stands for metal) photocatalysts and Pd‐modified‐photocatalysts (Table S4).

To identify the optimal cocatalyst, we screened various non‐noble metals (Cu, Co, Ni, Zn, Fe, and Mn) and noble metals (Pt, Rh, Pd, Ru, and Ir) as cocatalysts on N (30) ‐TiO_2_ with identical loading amounts (Figures S10 and S11). Among the noble metals, PdN‐TiO_2_ exhibits a superior H_2_ evolution rate, substantially outperforming the other noble and non‐noble metal counterparts. This remarkable enhancement underscores the intrinsic catalytic superiority of Pd active sites and the strong synergistic electronic coupling between Pd SAs and the doped N species. Synthesis condition optimization shows that 1% PdN‐TiO_2_ achieves the highest activity at 400°C calcination and stirring for 8 h (185.8 mmol g^−1^ h^−1^), beyond which performance declines due to structural degradation or non‐uniform dispersion (Figures S12 and S13).

Figure 3b further presents the photocatalytic H_2_ evolution rate profiles of samples with gradient Pd loadings (0.5 wt.%, 0.75 wt.%, 1 wt.%, 1.25 wt.%, and 1.5 wt.%). Corresponding time‑dependent H_2_ evolution curves for different Pd loading samples are shown in Figure S14. 1% PdN‐TiO_2_ achieves the maximum H_2_ production efficiency, while the control sample of 1% Pd‐TiO_2_ without N doping shows an obviously low H_2_ evolution rate. The decrease in H_2_ evolution rate observed when the Pd loading exceeds 1 wt.% is likely attributable to aggregation of Pd species, which both reduce light absorption by TiO_2_ support and impedes mass transfer [19]. TEM observations further confirm that obvious Pd aggregation occurs when Pd loading exceeds 1 wt.%. The formed Pd nanoparticles not only shield incident light and weaken photon absorption of TiO_2_ substrate, but also reduce the number of highly active Pd SAs sites, jointly resulting in the gradual decay of photocatalytic H_2_ production activity (Figure S15). Such a distinct difference directly confirms that the N‐induced asymmetric coordination environment creates high efficiency catalytic microenvironments for anchored Pd SAs.

Benefiting from the optimized electronic structure and favorable reaction kinetics, the 1% PdN‐TiO_2_ exhibits an ultrahigh H_2_ evolution rate up to 185.8 mmol g^−1^ h^−1^ (Figure 3c), substantially outperforming pristine TiO_2_ (0.64 mmol g^−1^ h^−1^), N (30)‐TiO_2_ (5.83 mmol g^−1^ h^−1^), and 1% Pd‐TiO_2_ (106.3 mmol g^−1^ h^−1^). Notably, AQE of 1% PdN‐TiO_2_ reaches 67.4% at 365 nm, which compares favorably with recently reported TiO_2_‐based SA photocatalysts (Table S4). The AQE values at 385, 400, and 420 nm are 30.1%, 5.3%, and 1.4%, respectively (Figure S16). The decrease in AQE with increasing wavelength is consistent with the UV–vis absorption spectrum of 1% PdN‐TiO_2_ (Figure 2d).

Cycling stability measurements demonstrate that no obvious attenuation of photocatalytic activity is observed after consecutive 15 h cycling tests, demonstrating excellent long‐term operational durability (Figure 3d). The positions and intensities of the characteristic XRD diffraction peaks of 1% PdN‐TiO_2_ before and after the cyclic reaction are consistent with those of the fresh catalyst (Figure S17a), with no new phases formed, confirming the stability of crystal structure during the cyclic reaction. The 0.35 nm lattice fringes corresponding to the (101) plane of TiO_2_ in the TEM image of the recycled sample is clearly observed in the high‐resolution region (Figure S17b), indicating the structural stability of the support. The Pd, N, Ti, and O elements mapping images of the recycled sample, presents their homogeneous distribution on the support surface without obvious element aggregation or leaching (Figure S17c). After 15 h of cycling, ICP‑OES analysis was further conducted the Pd loading drops from the initial 0.96 wt.% of fresh 1% PdN‑TiO_2_ to 0.89 wt.%. This result confirms partial Pd detachment from the catalyst surface, which would induce a certain degree of stability degradation. To further assess the chemical‐state evolution of the active sites upon photocatalytic cycling, XPS characterizations of 1% PdN‐TiO_2_ were performed before and after photocatalytic cycling. The Pd 3d, N 1s, Ti 2p, and O 1s spectra of the cycled sample are nearly identical to those of the fresh counterpart (Figure S17d), demonstrating that the chemical state of Pd is well preserved after cycling. These results collectively demonstrate that 1% PdN‐TiO_2_ catalyst exhibits excellent structural stability during the cyclic photocatalytic reaction.

In addition, a comprehensive performance comparison with recently reported TiO_2_‐based and Pd‐modified photocatalysts further highlights the prominent advantages of our 1% PdN‐TiO_2_ (Figure 3e). Overall, photocatalytic performance measurements reveal that N doping plays a critical role in regulating band alignment and promoting photon utilization than simple Pd loading. The co‐regulation strategy of N doping and Pd SA anchoring synergistically optimizes electronic structure, accelerates charge separation, and substantially boosts photocatalytic H_2_ evolution rate.

### 2.3 Photocatalytic Mechanistic Study

Figure 4a presents the steady‐state photoluminescence (PL) spectra collected to evaluate the photogenerated charge recombination behavior of the photocatalysts. Pristine TiO_2_ displays intense PL emission, which originates from severe intrinsic charge recombination and sluggish interfacial charge transfer kinetics. After the simultaneous incorporation of N dopants and Pd SAs, 1% PdN‐TiO_2_ exhibits a pronounced reduction in PL intensity, clearly demonstrating that the synergistic Pd‐N interaction effectively inhibits the recombination of photogenerated electron‐hole pairs. Figure 4b shows the time‐resolved photoluminescence (TRPL) spectra of different catalysts (Table S3). The average carrier lifetime of TiO_2_ is 3.57 ns, while N (30)‐TiO_2_ exhibits a prolonged lifetime of 4.60 ns due to the optimized lattice configuration. In contrast, 1% Pd‐TiO_2_ and 1% PdN‐TiO_2_ show significantly shortened average carrier lifetimes of 2.20 and 1.13 ns, respectively. The sharp decrease in fluorescence lifetime confirms that the synergistic interaction between N doping and Pd SAs enables rapid extraction and efficient separation of photogenerated charge carriers, thereby greatly promoting the photocatalytic reaction kinetics [28].

**FIGURE 4:**
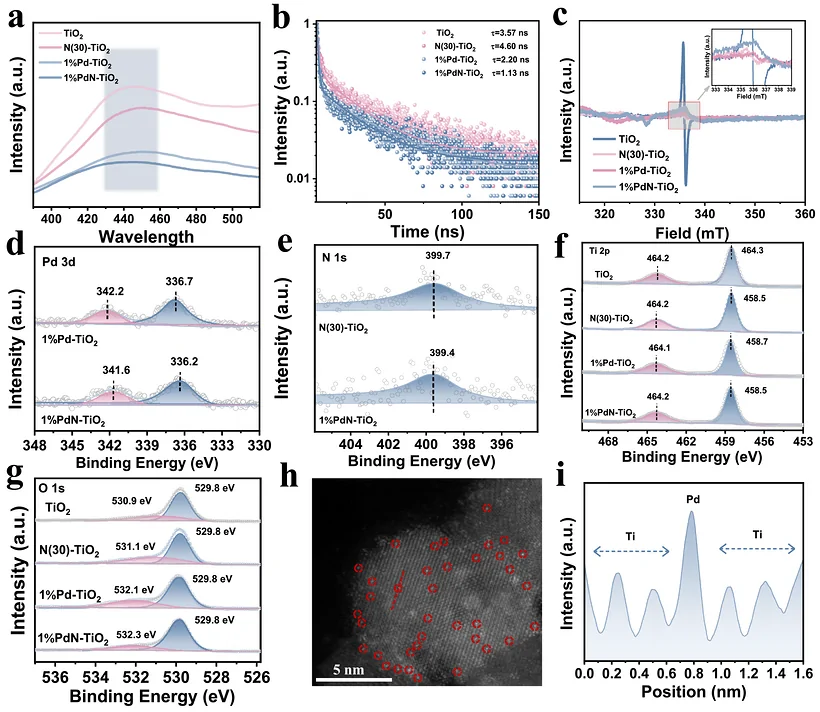
Optical and surface chemical characterizations of the photocatalysts. (a) PL, (b) TRPL, and (c) EPR of TiO_2_, N (30)‐TiO_2_, 1%Pd‐TiO_2_, and 1%PdN‐TiO_2_. XPS spectra of (d) Pd 3d for 1%Pd‐TiO_2_, and 1%PdN‐TiO_2_, (e) N 1s for 1%Pd‐TiO_2_, and 1%PdN‐TiO_2_, (f) Ti 2p for TiO_2_, N (30)‐TiO_2_, 1%Pd‐TiO_2_, and 1%PdN‐TiO_2_, (g) O 1s for TiO_2_, N (30)‐TiO_2_, 1%Pd‐TiO_2_, and 1%PdN‐TiO_2_. (h) HAADF‐STEM images of 1%PdN‐TiO_2_. (i) The corresponding atomic intensity profiles of Line 1 marked in (h).

The electron paramagnetic resonance (EPR) spectra show that pristine TiO_2_ exhibits a strong signal at ∼336 mT (Figure 4c), indicating abundant Ti^3+^/oxygen vacancy (O_v_) defects. Both N (30)‐TiO_2_ and 1% Pd‐TiO_2_ show much weaker signals, suggesting that single modification suppresses these intrinsic defects. In contrast, 1% PdN‐TiO_2_ displays a broadened weak signal (333–339 m T), implying new paramagnetic centers from N or Pd‐related species due to synergistic interactions. Overall, Pd─N co‐modulation effectively tunes the defect structure, introducing new electronic states while reducing original O_v_. All samples exhibit characteristic signals of Ti 2p, O 1s, and C 1s in the full‐scan XPS spectra (Figure S18), while the inset in Figure S18 presents 1% Pd‐TiO_2_ and 1% PdN‐TiO_2_ additionally show signals corresponding to Pd, as well as N (30)‐TiO_2_ and 1% PdN‐TiO_2_ display signals corresponding to N, confirming that N and Pd elements have been successfully doped and loaded onto TiO_2_ support.

The high‐resolution Pd 3d spectra demonstrate that both 1% Pd‐TiO_2_ and 1% PdN‐TiO_2_ exhibit typical characteristic peaks assigned to Pd^2+^ species (Pd 3d_5/2_ ≈ 336.5 eV, Pd 3d_3/2_ ≈ 341.8 eV, Figure 4d), while no characteristic signal corresponding to metallic Pd^0^ is detected, confirming that Pd is exclusively presented in the isolated SA oxidation state. Notably, the Pd 3d binding energy of 1% PdN‐TiO_2_ exhibits a distinct negative shift compared with 1% Pd‐TiO_2_, which originates from the lower electronegativity of N (3.04) relative to O (3.44), indicating a charge redistribution and electron transfer phenomenon [29, 30, 31]. The high‐resolution N 1s spectra further clarify the doping configuration of lattice N (Figure 4e), where both N (30)‐TiO_2_ and 1% PdN‐TiO_2_ display a dominant characteristic peak at approximately 399.7 and 399.4 eV, attributed to lattice‐integrated N species originating from the substitution of lattice oxygen by N heteroatoms. Additionally, the slight negative shift in 1% PdN‐TiO_2_ confirms the direct interfacial coordination between doped N and anchored Pd SAs rather than physical surface adsorption. Moreover, the Ti 2p high‐resolution spectra present a noticeable positive shift in binding energy after N doping (Figure 4f), revealing spontaneous electron transfer from Ti sites to adjacent N dopants and further consolidating the formation of stable lattice‐integrated Ti‐N structures. Meanwhile, there are two characteristic peaks corresponding to surface defect oxygen (about 530.9–532.3 eV) and lattice oxygen (about 529.8 eV) in the O 1s spectra (Figure 4g), with the latter gradually shifting to higher binding energy upon N and Pd doping, reflecting the modulation of surface electronic environments induced by the heteroatom doping and Pd SAs [32].

Aberration‐corrected high‐angle annular dark‐field scanning transmission electron microscopy (HAADF‐STEM) was further employed to visualize the atomic dispersion and local coordination environment. Figure 4h shows the atomic‐resolution HAADF‐STEM image of 1% PdN‐TiO_2_, where the uniformly isolated bright spots marked by red circles correspond to high‐Z Pd SAs [33], with no Pd clusters or nanoparticles being observed. Figure 4i presents the corresponding line‐scan elemental profile along the dashed line, with a distinct Pd signal peak clearly detected between Ti atomic columns, confirming that Pd atoms are located at Ti lattice sites.

### 2.4 Mechanisms of Metal‐Support Electronic Interaction

Figure 5a presents the in situ XPS Pd 3d spectra of 1% PdN‐TiO_2_ catalyst before and under illumination. All binding energies are calibrated using the C 1s peak at 284.8 eV. The characteristic peaks of Pd 3d orbitals are initially located at 336.72 eV (Pd 3d_5/2_) and 341.82 eV (Pd 3d_3/2_) [4], which are assigned to Pd^2+^ oxidation state. During irradiation, both peaks shifted significantly toward lower binding energies to 334.82 and 340.12 eV with shifts of 1.90 and 1.70 eV, respectively, and such a decrease in binding energy directly demonstrates a remarkable increase in the electron density of Pd sites [4, 34], confirming that Pd acts as an electron acceptor, serving as an enrichment center for photogenerated electrons under illumination. Correspondingly, the XPS N 1s peak is initially at 398.42 eV, corresponding to the initial chemical state of doped N, which shifts to higher binding energy at 399.72 eV with a shift of 1.30 eV upon irradiation (Figure 5b). The increase in binding energy indicates a reduction in the electron density of N atoms, validating that N doping modulates the electron density of adjacent Ti and O sites, facilitating directional electron transfer toward Pd. The in situ characteristic peaks of Ti 2p orbitals are initially located at 458.5 eV (Ti 2p_3/2_) and 464.3 eV (Ti 2p_1/2_) (Figure 5c). During irradiation, both peaks shift toward lower binding energies to 457.8 and 463.6 eV, indicating an increase in the electron density of Ti sites and confirming the effective transfer of photogenerated electrons in the support [35]. The opposite trends in binding energy shifts of Pd and N sites directly reveal the directional electron transfer from N to Pd during the photocatalytic process, providing direct experimental evidence for the superior photocatalytic performance of 1% PdN‐TiO_2_.

**FIGURE 5:**
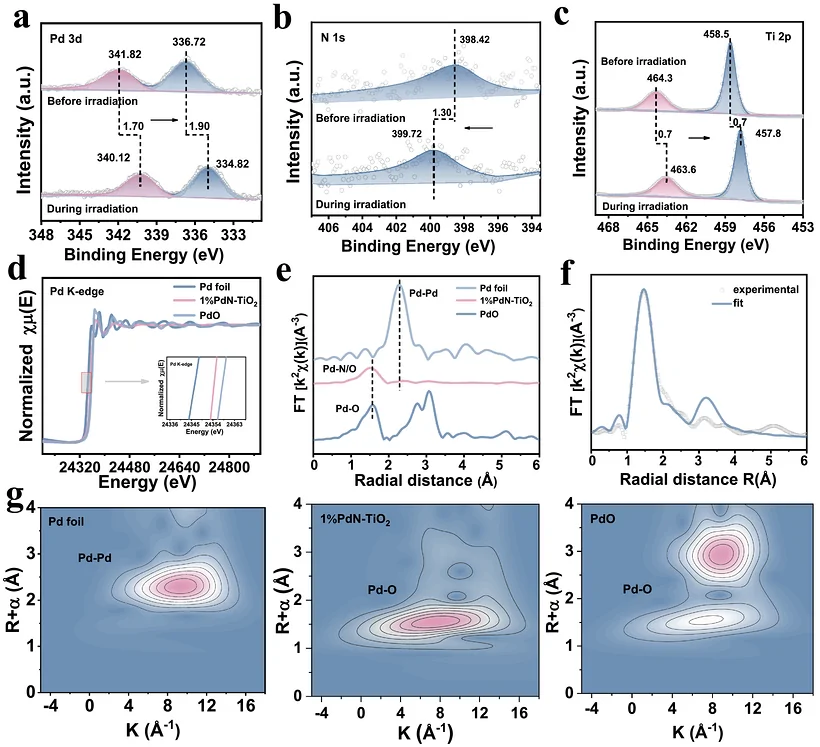
Electronic properties and local coordination environment of the photocatalysts. In situ XPS for (a) Pd 3d 1%PdN‐TiO_2_, (b) N 1s of 1%PdN‐TiO_2_, and (c) Ti 2p of 1%PdN‐TiO_2_. (d) Normalized Pd K‐edge XANES spectra of Pd foil, 1%PdN‐TiO_2_, and PdO. (e) FT‐EXAFS spectra of 1%PdN‐TiO_2_ and reference Pd foil and PdO. (f) Fitted Pd K3‐edge EXAFS spectra of K space for 1%PdN‐TiO_2_. (g) k^2^‐weighted wavelet transforms for the Pd K‐edge XAFS signals of Pd foil, 1%PdN‐TiO_2_, and PdO based on Morlet wavelets.

Figure 5d presents the Pd K‐edge X‐ray absorption near‐edge structure (XANES) spectra of 1% PdN‐TiO_2_, Pd foil, and crystalline PdO references, where the absorption edge position of 1% PdN‐TiO_2_ lies between those of metallic Pd and PdO, excluding the formation of Pd clusters and confirming the positively charged SA state of Pd [36]. There is only a dominant peak at ∼1.6 Å assigned to Pd─O/Pd─N coordination without any detectable Pd─Pd metallic bonding signal around 2.5 Å in the corresponding Fourier‐transformed extended X‐ray absorption fine structure (EXAFS) spectra (Figure 5e). The R‐space EXAFS fitting is in excellent agreement with the experimental data (Figure 5f), and quantitative least‐squares fitting based on a dual‐pathway model (Pd─O + Pd─N) (Table S3) reveals that Pd has an average Pd─O bond length of 2.03 Å with a coordination number of 2.5 and a Pd─N bond length of 2.08 Å with a coordination number of 1.2, while the K‐space fitting curve also well matches the experimental oscillations (Figure S19). Figure 5g presents the WT‐EXAFS contour plots of Pd foil, PdO, and 1% PdN‐TiO_2_. Pd foil exhibits obvious Pd─Pd metallic scattering signals at R ≈ 2.5 Å and K ≈ 8 Å^−1^, while PdO and 1% PdN‐TiO_2_ only show Pd─O/Pd─N coordination signals at R ≈ 1.6 Å and K ≈ 5 Å^−1^ without Pd─Pd signals, confirming the atomic dispersion of Pd [36, 37]. Collectively, these XAFS results unambiguously demonstrate that Pd species are atomically dispersed as isolated SAs on the TiO_2_ support, forming a unique Pd─N/O mixed coordination microenvironment.

The femtosecond transient absorption (TA) spectra of 1% Pd‐TiO_2_ show that the absorption signal at 340 nm decays rapidly with increasing delay time (Figure 6a), indicating ultrafast non‑radiative relaxation of photogenerated electrons from the conduction band to deep‑level defect states. The corresponding kinetic trace at 340 nm exhibits an extremely short carrier lifetime (Figure 6b), suggesting that photogenerated electron‐hole pairs undergo severe bulk recombination and cannot migrate effectively to the surface for catalytic reactions. In contrast, the TA spectra of 1% PdN‐TiO_2_ display distinctly different dynamics (Figure 6c). The kinetic trace at 340 nm shows a prolonged carrier lifetime (Figure 6d). This phenomenon is attributed to the synergistic effects between Pd SAs and N doping, wherein the asymmetric N─Pd─O coordination structure effectively captures and prolongs the lifetime of photogenerated electrons, thereby forming a long‑lived charge‑separated state [38].

**FIGURE 6:**
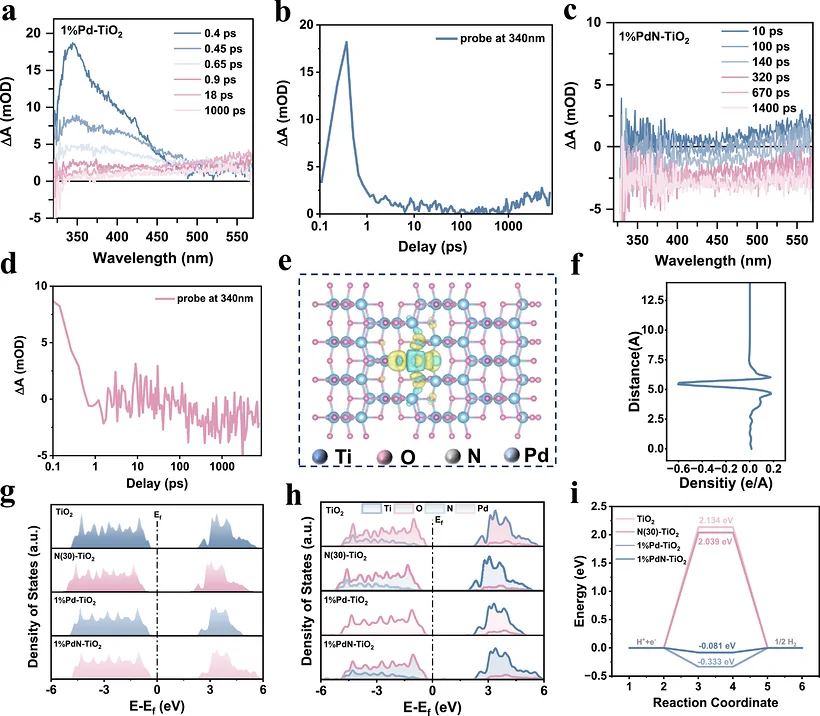
Photocatalytic mechanisms and DFT calculation results. (a) TA signals of 1%Pd‐TiO_2_, (b) Transient absorption spectra of 1%Pd‐TiO_2_ probed at 340 nm, (c) TA signals of 1%PdN‐TiO_2_, (d) 1%PdN‐TiO_2_ probed at 340 nm. (e, f) Charge density difference maps of 1%PdN‐TiO_2_. (g) Total DOS. (h) Element‐projected LDOS, (i) Free energy diagrams of H* adsorption on the surface of TiO_2_, N (30)‐TiO_2_, 1%Pd‐TiO_2_, and 1%PdN‐TiO_2_.

Figure S20 presents the geometrically optimized models obtained via DFT calculations. In 1% PdN‐TiO_2_ model, the first coordination shell of Pd contains three O atoms and one N atom, which is consistent with the synchrotron radiation test results. Figure S21 further displays the optimized H* adsorption configurations on the different active sites of TiO_2_, N (30)‐TiO_2_, 1% Pd‐TiO_2_, and 1% PdN‐TiO_2_, which serve as the initial structures for the subsequent HER free‐energy calculations. Figure 6e shows the differential charge density distribution of 1% PdN‐TiO_2_, where the yellow regions represent charge accumulation, intuitively revealing the electron delocalization between N‐doped sites and Pd‐anchored sites, confirming obvious charge transfer at the Pd‐N coordination interface [15, 39, 40]. The one‐dimensional charge density distribution along the Pd atom direction, further quantifies the charge accumulation state at the Pd sites (Figure 6f).

Total density of states (TDOS) comparisons of TiO_2_, N (30)‐TiO_2_, 1% Pd‐TiO_2_ and 1% PdN‐TiO_2_ reveal that 1% PdN‐TiO_2_ introduces a remarkable abundance of mid‐gap electronic states close to the Fermi level (E_f_) (Figure 6g). Local density of states (LDOS) further verifies that these newly emerged mid‐gap states primarily originate from strong orbital hybridization between Pd 4d and N 2p electrons, which effectively modifies the electronic structure of the TiO_2_ matrix [41] (Figure 6h).

The HER free‐energy profiles in Figure 6i quantitatively confirm this electronic modulation effect: TiO_2_ possesses a high reaction energy barrier of 2.134 eV, while single N doping only slightly decreases the barrier to 2.039 eV. In contrast, the Pd─N co‐modified 1% PdN‐TiO_2_ delivers a much lower Gibbs free energy of −0.333 eV for H* adsorption, which significantly deviates from the −0.081 eV observed for monometallic 1% Pd‐TiO_2_. This substantial shift indicates that the asymmetric N─Pd─O coordination effectively weakens the excessively strong H* binding on Pd sites, thereby modulating the H* adsorption‐desorption balance. Such results clearly demonstrate that the N─Pd─O asymmetric coordination configuration not only effectively modulates the Gibbs free energy for H* adsorption but also substantially boosts the photocatalytic H_2_ evolution performance [42, 43].

## 3 Conclusions

In summary, we demonstrate that anchoring Pd SAs onto N‐doped MOF‐derived TiO_2_ creates an asymmetric N─Pd─O coordination environment that synergistically enhances photocatalytic H_2_ evolution. The optimized 1% PdN‐TiO_2_ catalyst achieves a high H_2_ evolution rate up to 185.8 mmol g^−1^ h^−1^ with an apparent quantum efficiency of 67.4% at 365 nm, outperforming the undoped counterpart. Combined in situ spectroscopic and theoretical analyses reveal that the asymmetric coordination suppresses charge recombination, enables directional electron transfer from N to Pd under illumination, and lowers the H_2_ evolution rate barrier via Pd 4d and N 2p hybridization. More broadly, this work highlights heteroatom‐induced asymmetric coordination as a general strategy for tuning charge dynamics and surface reactivity in SA photocatalysts.

## Author Contributions

Y.S. and Z.L.W. supervised the project. Y.X.Y., Z.L.W., and G.R.J. designed the studies and wrote the paper. Y.X.Y. and W.H.L. synthesized the catalysts and performed the catalytic tests. Y.X.Y. and G.R.J. conducted and analyzed the structural characterizations. G.R.J. and Z.L.W. revised and polished the manuscript. All authors discussed the results and commented on the manuscript.

## Conflicts of Interest

The authors declare no conflicts of interest.

## Supporting information




**Supporting File**: advs78590‐sup‐0001‐SuppMat.docx.

## Data Availability

The data that support the findings of this study are available from the corresponding author upon reasonable request.

## References

[1] Y. Li , H. Zhou , S. Cai , et al., “Electrolyte‐Assisted Polarization Leading to Enhanced Charge Separation and Solar‐to‐Hydrogen Conversion Efficiency of Seawater Splitting,” Nature Catalysis 7, no. 1 (2024): 77–88, 10.1038/s41929-023-01069-1.

[2] C. Wang , K. Wang , Y. Feng , et al., “Co and Pt Dual‐Single‐Atoms With Oxygen‐Coordinated Co–O–Pt Dimer Sites for Ultrahigh Photocatalytic Hydrogen Evolution Efficiency,” Advanced Materials 33, no. 13 (2021): 2003327, 10.1002/adma.202003327. 33615589

[3] L. Wang , H. Xiao , L. Yang , J. Li , J. Zi , and Z. Lian , “Hollow Nanobox‐Shaped Cu 2‐x S@Zn x Cd 1‐x S Heterojunction by Light Multireflection With Z‐Scheme Mechanism for Enhanced Photocatalytic Hydrogen Production,” Advanced Functional Materials 35, no. 9 (2024): 2416358, 10.1002/adfm.202416358.

[4] Y. Tang , Z.‐F. Xu , Y. Sun , et al., “Simultaneous and Efficient Utilization of Photogenerated Electrons and Holes: A Case of Single‐Atom Pd‐Anchored CDS Twins,” Energy & Environmental Science 17, no. 20 (2024): 7882–7894, 10.1039/D4EE03092C.

[5] B. Yan , D. Liu , X. Feng , M. Shao , and Y. Zhang , “Ru Species Supported on MOF‐Derived N‐Doped TiO _2_ /C Hybrids as Efficient Electrocatalytic/Photocatalytic Hydrogen Evolution Reaction Catalysts,” Advanced Functional Materials 30, no. 31 (2020): 2003007, 10.1002/adfm.202003007.

[6] G. Egea , J. Santiago Martinez , M. A. Molina , et al., “In Situ Amine Formation to Modulate MOF‐Derived PdIn N‐Doped Carbon Catalysts,” Advanced Materials 38, no. 51 (2025): 15111, 10.1002/adma.202515111. PMC13569077 41267455

[7] Z.‐T. Chen , J.‐T. Lee , and M.‐Y. Wey , “Fabrication of Visible‐Light‐Driven Bimetallic MOF‐Derived Ag/NiO_x_/N‐TiO_2_: Photocatalytic Hydrogen Production Mechanism and Methanol Transformation Pathway,” Journal of Environmental Chemical Engineering 10, no. 5 (2022): 108375, 10.1016/j.jece.2022.108375.

[8] R. Zhang , J. Shi , L. Fu , et al., “Direct Photocatalytic Methane Oxidation to Formaldehyde by N Doping Co‐Decorated Mixed Crystal TiO _2_ ,” ACS Nano 18, no. 20 (2024): 12994–13005, 10.1021/acsnano.4c01318. 38721844

[9] Z. Tao , B. Zhang , W. Chen , R. Cao , J. Liu , and L. Wang , “Facile Construction of N‐Doped TiO_2_ Nanohybrids With Enriched Oxygen Vacancies for Enhanced Photocatalytic Green Hydrogen Production,” Ceramics International 51, no. 19 (2025): 27971–27981, 10.1016/j.ceramint.2025.04.011.

[10] X. Fang , Q. Shang , Y. Wang , et al., “Single Pt Atoms Confined Into a Metal–Organic Framework for Efficient Photocatalysis,” Advanced Materials 30, no. 7 (2018): 1705112, 10.1002/adma.201705112. 29315871

[11] R. Sun , X. Cao , J. Ma , et al., “Regulation of Pd Single‐Atom Coordination for Enhanced Photocatalytic Oxidation of Toluene to Benzaldehyde,” Nature Synthesis 4, no. 8 (2025): 965–975, 10.1038/s44160-025-00782-y.

[12] J. H. Kim , S. Wu , L. Zdrazil , N. Denisov , and P. Schmuki , “2D Metal–Organic Framework Nanosheets based on Pd‐TCPP as Photocatalysts for Highly Improved Hydrogen Evolution,” Angewandte Chemie International Edition 63, no. 7 (2024): 202319255, 10.1002/anie.202319255. 38157446

[13] T. Wang , X. Tao , X. Li , K. Zhang , S. Liu , and B. Li , “Synergistic Pd Single Atoms, Clusters, and Oxygen Vacancies on TiO _2_ for Photocatalytic Hydrogen Evolution Coupled With Selective Organic Oxidation,” Small 17, no. 2 (2020): 2006255, 10.1002/smll.202006255. 33325647

[14] Z. Wei , Z. Zhao , C. Qiu , et al., “Tripodal Pd Metallenes Mediated by Nb_2_C MXenes for Boosting Alkynes Semihydrogenation,” Nature Communications 14, no. 1 (2023): 661, 10.1038/s41467-023-36378-3. PMC9905561 36750563

[15] B. Xiao , C. Shen , Z. Luo , et al., “Modulating d‐p‐Band Electron Coupling by N‐Doping for Promoting CuSAs‐N/TiO_2_ Photocatalytic Hydrogen Evolution,” Chemical Engineering Journal 499 (2024): 156331, 10.1016/j.cej.2024.156331.

[16] X. Zhao , K. Gao , S. Xue , W. Ran , and R. Liu , “Synergistic Effects of Oxygen Vacancies and Pd Single Atoms on Pd@TiO_2_− for Efficient HER Catalysis,” Chinese Chemical Letters 36, no. 6 (2025): 110309, 10.1016/j.cclet.2024.110309.

[17] M. Dan‐Hardi , C. Serre , T. Frot , et al., “A New Photoactive Crystalline Highly Porous Titanium(IV) Dicarboxylate,” Journal of the American Chemical Society 131, no. 31 (2009): 10857–10859, 10.1021/ja903726m. 19621926

[18] Y. Zhang , C. X. Harris , P. Wallenmeyer , J. Murowchick , and X. Chen , “Asymmetric Lattice Vibrational Characteristics of Rutile TiO _2_ as Revealed by Laser Power Dependent Raman Spectroscopy,” The Journal of Physical Chemistry C 117, no. 45 (2013): 24015–24022, 10.1021/jp406948e.

[19] H. Wang , H. Qi , X. Sun , et al., “High Quantum Efficiency of Hydrogen Production From Methanol Aqueous Solution With PtCu–TiO_2_ Photocatalysts,” Nature Materials 22, no. 5 (2023): 619–626, 10.1038/s41563-023-01519-y. 37037960

[20] J. Jin , C. Wang , X.‐N. Ren , et al., “Anchoring Ultrafine Metallic and Oxidized Pt Nanoclusters on Yolk‐Shell TiO_2_ for Unprecedentedly High Photocatalytic Hydrogen Production,” Nano Energy 38 (2017): 118–126, 10.1016/j.nanoen.2017.05.025.

[21] Z. S. Huang , Y. F. Wang , M. Y. Qi , M. Conte , Z. R. Tang , and Y. J. Xu , “Interface Synergy of Exposed Oxygen Vacancy and Pd Lewis Acid Sites Enabling Superior Cooperative Photoredox Synthesis,” Angewandte Chemie International Edition 63, no. 47 (2024): 202412707, 10.1002/anie.202412707. 39136931

[22] X. Chen , X. Zhang , Y.‐H. Li , et al., “Transition Metal Doping BiOBr Nanosheets With Oxygen Vacancy and Exposed {102} Facets for Visible Light Nitrogen Fixation,” Applied Catalysis B: Environmental 281 (2021): 119516, 10.1016/j.apcatb.2020.119516.

[23] H. Hu , X. Zhang , K. Zhang , et al., “Construction of a 2D/2D Crystalline Porous Materials Based S‐Scheme Heterojunction for Efficient Photocatalytic H _2_ Production,” Advanced Energy Materials 14, no. 11 (2024): 2303638, 10.1002/aenm.202303638.

[24] J. Zhang , P. Qiao , H. Tan , et al., “Species Heterogeneity and Synergy to Boost Photocatalytic Hydrogen Evolution,” ACS Catalysis 14, no. 20 (2024): 15699–15712, 10.1021/acscatal.4c03593.

[25] J. Xiao , J. J. M. Vequizo , T. Hisatomi , et al., “Simultaneously Tuning the Defects and Surface Properties of Ta _3_ N _5_ Nanoparticles by Mg–Zr Codoping for Significantly Accelerated Photocatalytic H _2_ Evolution,” Journal of the American Chemical Society 143, no. 27 (2021): 10059–10064, 10.1021/jacs.1c04861. 34196527

[26] W. Li , G. Zuo , S. Ma , et al., “Localized Photothermal Effect Mediated Hollow S‐Scheme NiCo_2_O_4_@ ZnIn_2_S_4_ for Enhanced Photocatalytic Hydrogen Evolution,” Applied Catalysis B: Environment and Energy 365 (2025): 124971.

[27] Q. Shen , C. Dong , S. Feng , X. Zhang , Q. Li , and J. Xue , “The Synergistic Effect of Non‐Compensated Cu/N Co‐Doping and Graphene Enhances the Dual‐Channel Generation of H_2_O_2_ Over TiO_2_ Photocatalysts,” Chinese Journal of Catalysis 78 (2025): 252–264, 10.1016/S1872-2067(25)64799-1.

[28] M. S. Nasir , Y. Zhao , H. Ye , et al., “Efficient Hole Extraction and *OH Alleviation by Pd Nanoparticles on GaN Nanowires in Seawater for Solar‐Driven H _2_ and H _2_ O _2_ Generation,” Angewandte Chemie International Edition 64, no. 10 (2025): 202420796, 10.1002/anie.202420796. 39891897

[29] H. Wang , Y. Qin , Y. Wu , et al., “Anti‐Phage Defense Mechanism Involving Phage‐Encoded Dna Binding Protein and Bacterial Reverse Transcriptase DRT4,” Nature Communications 17, no. 1 (2024): 0289, 10.1038/s41467-025-66997-x. PMC12783706 41330908

[30] S. K. M. Padavala and K. A. Stoerzinger , “Role of Hydride Formation in Electrocatalysis for Sustainable Chemical Transformations,” ACS Catalysis 13, no. 7 (2023): 4544–4551, 10.1021/acscatal.3c00204.

[31] L. Zhang , F. Zhang , H. Xue , et al., “Mechanism Investigation of PtPd Decorated Zn_0. 5_Cd_0. 5_S Nanorods With Efficient Photocatalytic Hydrogen Production Combining With Kinetics and Thermodynamics,” Chinese Journal of Catalysis 42, no. 10 (2021): 1677.

[32] Y. Liu , P. Wang , L. Xie , et al., “Electronic Metal‐Support Interactions Boost *OOH Intermediate Generation in Cu/In _2_ Se _3_ for Electrochemical H _2_ O _2_ Production,” Angewandte Chemie International Edition 63, no. 23 (2024): 202319470, 10.1002/anie.202319470. 38566301

[33] Y. Zhang , J. Zhao , H. Wang , et al., “Single‐Atom Cu Anchored Catalysts for Photocatalytic Renewable H_2_ Production With a Quantum Efficiency of 56%,” Nature Communications 13, no. 1 (2022): 58, 10.1038/s41467-021-27698-3. PMC8748625 35013219

[34] T. Lv , B. Xiao , F. Xia , et al., “Insights Into Synergistic Effect of Pd Single Atoms and Sub‐Nanoclusters on TiO_2_ for Enhanced Photocatalytic H_2_ Evolution,” Chemical Engineering Journal 450 (2022): 137873, 10.1016/j.cej.2022.137873.

[35] J. Xu , D. Gao , H. Yu , et al., “Palladium‐Copper Nanodot as Novel H_2_‐Evolution Cocatalyst: Optimizing Interfacial Hydrogen Desorption for Highly Efficient Photocatalytic Activity,” Chinese Journal of Catalysis 43, no. 2 (2022): 215–225, 10.1016/S1872-2067(21)63830-5.

[36] J. Xie , J. Cheng , J. Peng , et al., “A Tandem Catalysis for Isoindolinone Synthesis Over Single‐Atom Pd/TiO _2_ Catalyst,” Angewandte Chemie International Edition 64, no. 3 (2024): 202415203, 10.1002/anie.202415203. 39263728

[37] Q. Shi , R. Bai , C. Zhang , et al., “Facet‐Engineered Anatase TiO _2_ Nanoflowers With Anchored Single‐Atom Pd for Ultrafast Hydrogen Storage Kinetics,” Advanced Energy Materials 16, no. 7 (2025): 05141, 10.1002/aenm.202505141.

[38] C. Li , P. Zhang , M. Zheng , et al., “Hydroxylated Rh Single‐Atom Antennas Assembled on Carbon Nitride Toward Stable Photocatalytic Hydrogen Evolution,” Advanced Science 13, no. 6 (2025): 18847, 10.1002/advs.202518847. PMC12866703 41399990

[39] W. Fu , Z. Geng , B. Jiang , et al., “Engineering Plasmon‐Semiconductor Coupling in Spatially Ordered Supraparticles for Boosted Photocatalytic Hydrogen Evolution,” Angewandte Chemie International Edition 65 (2026): 8359659. 10.1002/anie.8359659 42037131

[40] H. Yang , C. Ai , Y. Zhou , B. He , J. Zhang , and J. Yu , “Probing the LSPR Effect of Cu‐Doped ZnO Photocatalyst by Near‐Infrared Femtosecond Transient Absorption Spectroscopy,” Artificial Photosynthesis 2, no. 1 (2025): 53–66, 10.1021/aps.5c00021.

[41] B. Jia , J. Zhang , X. Chen , et al., “Electronic Structure Modulation of Nb _2_ O _5_ by Ru Single Atoms Enabling Efficient Hydrogen Storage of Magnesium Hydrides,” Angewandte Chemie International Edition 64, no. 33 (2025): 202511139, 10.1002/anie.202511139. 40511859

[42] H. Hu , X. Li , K. Zhang , et al., “Dual Modification of Metal–Organic Frameworks for Exceptional High Piezo‐Photocatalytic Hydrogen Production,” Advanced Materials 37, no. 20 (2025): 2419023, 10.1002/adma.202419023. PMC12087703 40159815

[43] X. Yang , N. Yang , Z. Qiu , et al., “Cu Surface Plasmon Resonance Promoted Charge Transfer in S‐Scheme System Enhanced Visible Light Photocatalytic Hydrogen Evolution,” Journal of Colloid and Interface Science 679 (2025): 710–722, 10.1016/j.jcis.2024.10.087. 39476625

