# Supplementary material for: Anchoring Pd Single Atoms on N‐Doped MOF‐Derived TiO2 for Photocatalytic Hydrogen Evolution With an Ultra‐High Rate up to 185.8 mmol g−1 h−1
Source: Adv Sci (Weinh). 2026 Nov 9:e78590. Online ahead of print. doi: 10.1002/advs.78590 (PMC13747895; doi:10.1002/advs.78590)
Supplement: Supplementary file 1 [file ADVS-9999-e78590-s001.docx]

**Anchoring Pd Single Atoms on N-Doped MOF-Derived TiO_2_ for Photocatalytic Hydrogen Evolution with an Ultra-high Rate up to 185.8 mmol g^-1^ h^-1^**

Xingyan Yang^a,b^, Guangri Jia^a^, Wenhao Li^a,b^, Mingzhu Xie^a^, Yong Shuai^a,b*^, Zhaolong Wang^a,b*^

*^a^ School of Energy Science and Engineering, Harbin Institute of Technology, Harbin 150001, P. R. China*

*^b^ Zhengzhou Advanced Research Institute of Harbin Institute of Technology, Zhengzhou 450046, P. R. China*

**Corresponding authors. E-mail addresses:* shuaiyong@hit.edu.cn (Y.S.); wangzhaolong@hit.edu.cn (Z.W.)

**Contents**

1. Methods

1.1. Materials and reagents

1.2. Preparation of photocatalyst samples

2. Computational details

3. Characterizations

4. Photocatalytic property evaluation

5. Photo-electrochemical measurements

6. Supplementary Figures

7. Supplementary Tables

8. References

**1. Methods**

**1.1 Materials and reagents**

Titanium butoxide (TBOT, ≥99%), 1,4-benzenedicarboxylic acid (PTA, ≥99%), N, N-Dimethylformamide (DMF, ≥99.5%), Palladium nitrate hydrate (Pd(NO_3_)_2_·xH_2_O, ≥99%), Rhodium chloride hydrate (RhCl_3_·3H_2_O, ≥98%), Ruthenium chloride hydrate (RuCl_3_·xH_2_O), Iridium chloride (IrCl_3_, ≥99.8%), Chloroplatinic acid hydrate (H_2_PtCl_6_·6H_2_O, AR), Nickel nitrate hexahydrate (Ni(NO_3_)_2_·6H_2_O, ≥98%), Iron nitrate nonahydrate (Fe(NO_3_)_3_·9H_2_O, AR), Ruthenium chloride hydrate (RuCl_3_·xH_2_O), Zinc chloride (ZnCl_2_, ≥98%), Manganese acetate ((CH_3_COO)_2_Mn, ≥98%), Cobalt nitrate hexahydrate (Co(NO_3_)_2_·6H_2_O, ≥99%), Na_2_SO_4_ (≥99.0%), CO(NH_2_)_2_ (≥99%), Methanol (anhydrous grade, ≥99.8%) were provided by Alad. Chem. RT Co. Ltd, (Shanghai, China). Ethanol (analytical grade, ≥99.5%) were provided by Tianjin Zhiyuan Chemical Reagent Co. Ltd, (Tianjin, China).

**1.2 Preparation of photocatalyst samples**

TiO_2_ sample was prepared according to the method reported in previous studies[1]. The specific process is as follows: 0.2 g of TiO_2_ was thoroughly mixed with 10 mg, 20 mg, 30 mg, 40 mg, and 50 mg of CO(NH_2_)_2_, respectively. The mixture was transferred into a 50 mL Teflon‑lined stainless‑steel autoclave, then, N doping was achieved through a hydrothermal reaction at a temperature of 180 ℃ for 6 h. After the reaction, the product was washed 3 times respectively with DI and ethanol, and dried at 60 ℃ overnight. The obtained powder was annealed at 350 ℃ in air for 2 h and ground to obtain N/TiO_2_ samples. The sample without N doping was referred as pristine TiO_2_. According to the amount of CO(NH_2_)_2_ added, the resulting samples were denoted as N(*x*)-TiO_2_ (*x* = 10, 20, 30, 40, 50), where *x* represents the amount of CO(NH_2_)_2_ added in mg. Afterwards, N (30) -TiO_2_ with the best performance was selected as the carrier, then Pt, Rh, Pd, Ru, Ir and other metal salts with the same mass ratio were loaded for comparative experiments. Through catalytic performance testing, Pd was selected as the optimal metal. Subsequently, Pd salt was used as the research object to regulate the addition amount of Pd (NO_3_)_2_ · *x*H_2_O and explore the influence of different Pd loading amounts. Correspondingly, the required amounts of the Pd(NO_3_)_2_·xH_2_O precursor (molecular weight = 230.43) for samples with Pd loadings of 0.5 wt%, 0.75 wt%, 1.0 wt%, 1.25 wt%, and 1.5 wt% were determined to be 2.71 mg, 4.06 mg, 5.41 mg, 6.77 mg, and 8.12 mg, respectively, based on 0.25 g of the N(30)-TiO_2_ support. After stirring for 8 h, the sample was annealed at 400 ℃ in air for 4 h to obtain *y*% PdN-TiO_2_ particles, where *y* denotes the theoretical mass fraction of Pd with values of 0.5, 0.75, 1, 1.25 and 1.5, respectively.

**2. Computational details**

All the calculations are performed in the framework of the density functional theory with the projector augmented plane-wave method, as implemented in the Vienna ab initio simulation package[2]. The generalized gradient approximation proposed by Perdew, Burke, and Ernzerhof is selected for the exchange-correlation potential[3]. The long range van der Waals interaction is described by the DFT-D3 approach[4]. The cut-off energy for plane wave is set to 400 eV. The energy criterion is set to 10^-6^ eV in iterative solution of the Kohn-Sham equation. A vacuum layer of 15 Å is added perpendicular to the sheet to avoid artificial interaction between periodic images. The Brillouin zone integration is performed using a 4×4×1 k-mesh. All the structures are relaxed until the residual forces on the atoms have declined to less than 0.05 eV/Å.

**3. Characterizations**

The structural, morphological, and surface properties of the catalysts were comprehensively characterized using a combination of techniques. X-ray diffraction (Aeris 600W, Cu Kα radiation) was employed to determine the crystallinity, and Raman spectra were collected with a LabRAM HR Evolution spectrometer (XperRam Compact). Morphology and microstructure were observed by TEM (JEM-2100), with aberration-corrected HAADF-STEM imaging performed on a Titan Cubed Themis G2 300 (Thermo Fisher). Optical absorption was analyzed by UV-vis diffuse reflectance spectroscopy (Metash UV-9000S, BaSO_4_ as reference). The surface area and porosity were measured from N_2_ adsorption-desorption isotherms at 77 K using a BET analyzer (3H-2000PS2). Paramagnetic centers were examined by electron paramagnetic resonance (EPR, Bruker EMXnano). Surface chemical states were investigated by PLXPS (Thermo ESCALAB 250Xi, Al Kα), and elemental composition was determined by ICP-OES (Agilent 7700X). Synchrotron-based X-ray absorption spectroscopy was carried out to analyze Pd K3-edge (BL11B beamline, SSRF, Si (311) monochromator) EXAFS and XANES spectra. The charge-carrier dynamics were further investigated by steady-state PL (Shimadzu FL-4500, excitation 365 nm) and time-resolved PL (FLS 1000, Edinburgh Instruments, excitation 365 nm, emission 430 nm).

Here, the bandgaps of the samples are calculated by the K-M method on the basis of the UV-vis diffuse absorption spectra by the following equation [5]

$\alpha h\nu=A\left(\text{h}\nu-E_{g}\right)^{1/2}$

where $\alpha$, $h\nu$, and $E_{\text{g}}$, $E_{\text{g}}$ are the absorption coefficient, the photon energy, and the direct bandgap, respectively. *A* is a constant.

**4. Photocatalytic property evaluation**

Photocatalytic H_2_ evolution experiments were conducted using a multichannel photochemical system (PCX-50C, Perfect Light Ltd.). Typically, 5 mg of catalyst was dispersed in 30 mL of DI/methanol (1:2, v/v) in a sealed quartz vessel, ultrasonicated, and purged with Ar (20 cycles). The suspension was irradiated with a 365 nm LED lamp (5 W) at 40 °C under continuous stirring. The evolved H_2_ was quantified at 30 min intervals via gas chromatography (F60, TCD detector, Ar carrier gas). After each cycle, the accumulated H_2_ was purged, and the measurement was repeated three times to obtain the average rate. The AQE was determined under monochromatic LED irradiation at 365, 385, 400, and 420 nm. In a typical test, 5 mg of catalyst was used under the same conditions as the photocatalytic H_2_ evolution experiment. The AQE is calculated using the following equation[6]:

$$AQE=\frac{2MN_{A}hc}{AI\text{t}\lambda}\times100\%$$

where *M* is the amount of evolved H_2_ (mol), *N*_A_ is Avogadro’s constant, *h* is Planck’s constant, *c* is the speed of light, *I* is the incident light intensity (32.8 mW cm^-2^, measured by a calibrated optical power meter, averaged value obtained by the thirteen‑point measurement method), *A* is the irradiation area (0.07065 cm^2^), *t* is the reaction time, and *λ* is the irradiation wavelength. The original three independent measurements refer to replicate tests using one batch of catalyst.

**5. Calculation of donor carrier density (N_d_) from Mott‑Schottky plots**

The Nd of n‑type semiconductor is derived from the Mott‑Schottky equation:

$N_{\text{d}}=\frac{2}{e\varepsilon\varepsilon_{0}A^{2}}\left[\frac{d\left(1/C^{2}\right)}{dE}\right]^{-1}$

Where$N_{\text{d}}$ is donor carrier density (cm^-3^), $e$is elementary charge, $1.602\times10^{-19}C$, $\varepsilon$is relative dielectric constant of TiO_2_, $\varepsilon_{0}$is permittivity of vacuum, $8.854\times10^{-14}F\cdot cm^{-1}$, $A$is geometric area of working electrode $\left(cm^{2}\right)$, $d\left(1/C^{2}\right)$ is the slope obtained from the linear region of Mott‑Schottky plot $F^{-2}\cdot V^{-1}$. All calculated E_fb_ and N_d_ values of the four samples are summarized in **Table S2**.

**6. Photo-electrochemical measurements**

Photoelectrodes were prepared by drop-casting a catalyst dispersion (5 mg in 5 wt.% Nafion/ethanol) onto FTO glass (2.0 × 2.5 cm^2^), followed by drying at 60 °C for 3 h. Photoelectrochemical (PEC) measurements were carried out on a CHI-660E workstation using a three-electrode cell, comprising the as-prepared film as the working electrode, Ag/AgCl as the reference, and Pt foil as the counter electrode. A 0.20 M Na_2_SO_4_ aqueous solution served as the electrolyte and was kept in a sealed amber polypropylene container at room temperature in the dark. Electrochemical impedance spectroscopy (EIS) was performed with a 5.0 mV AC perturbation over 10^5^-0.1 Hz. Under chopped illumination from a 150 W Xe lamp (94.7 mW cm^-2^), photocurrent responses were recorded via amperometric I-t measurements, while transient photocurrent data were acquired at 1.0 kHz with a 5.0 mV AC bias.

**7. Isotopic experiment.** The isotopic experiment was performed using a multi channel photochemical reactor under 365 nm LED irradiation. 5mg of 1%PdN-TiO_2_ was added into the solution of 10 mL of water and 20 mL of methanol, and irradiated for 2 h. Two separate experiments were carried out under the same conditions except for the deuterated part (D_2_O/CH_3_OH). Finally, the products were identified by Mass spectrum (MS, Hiden HPR-20).

**Supplementary Figures**

**Fig. S1.** XRD pattern of MIL-125.

**Fig. S2.** XRD patterns of PdN-TiO_2_ with different **a** N contents and **b** Pd contents (wt.%), respectively.

**Fig. S3.** ICP-OES analysis of 1%PdN-TiO_2_.

**Fig. S4.** The Fourier Transform Infrared Spectroscopy spectra for TiO_2_, N(30)-TiO_2,_ 1%Pd-TiO_2,_ 1%PdN-TiO_2_.

**Fig. S5.** BET tests of nitrogen adsorption and desorption isotherms of **a** TiO_2_. **b** N (30)-TiO_2_. **c** 1%Pd-TiO_2._ **d** 1%PdN-TiO_2_.

**Fig. S6.** BET tests of pore width distribution of **a** TiO_2_. **b** N(30)-TiO_2_. **c** 1%Pd-TiO_2._ **d** 1%PdN-TiO_2_.

**Fig. S7.** Photocatalytic H_2_ evolution rates of 1%PdN-TiO_2_ with different methanol to water ratio.

**Fig. S8.** Photocatalytic H_2_, HD, and D_2_ evolution curves of 1%PdN-TiO_2_ in the mixed solution of _a_ D_2_O/CH_3_OH and **b** H_2_O/CD_3_OD.

The D_2_O/CH_3_OH system, D_2_ is produced predominantly under illumination, with HD formed as a secondary product, whereas the H₂ level stays essentially constant. The H_2_O/CD_3_OD system behaves differently: H₂ is the main product, only a minor amount of HD is generated, and the D_2_ amount shows virtually no variation. These observations suggest that during photocatalysis, protons originate primarily from water, with methanol serving as a partial contributor. Moreover, the H_2_ evolution rate measured in H_2_O/CD_3_OD is markedly greater than the D_2_ evolution rate in D_2_O/CH_3_OH, a difference that can be explained by the faster decomposition kinetics of H₂O relative to D_2_O.

**Fig. S9.** Photocatalytic H_2_ evolution rates of 1%PdN-TiO_2_ with different temperatures.

**Fig. S10.** H_2_ evolution rate of different non-noble metals as cocatalyst.

**Fig. S11.** H_2_ evolution rate of different noble metals as cocatalyst.

**Fig. S12.** H_2_ evolution rate of different temperatures for 1%PdN-TiO_2_ synthesis.

**Fig. S13.** H_2_ evolution rates of 1%PdN-TiO_2_ at different stirring times.

**Fig. S14.** Photocatalytic H_2_ evolution rates of Pd-TiO_2_ and PdN-TiO_2_ with different Pd contents.

**Fig. S15.** TEM image element mappings of Pd, N, Ti and O of 1.25%PdN-TiO_2_.

**Fig. S16.** AQE for H_2_ evolution of 1%PdN-TiO_2_.

**Fig. S17. a** XRD patterns (before and after reaction). **b** TEM image **c** element mappings of Pd, N, Ti and O of 1%PdN-TiO_2_ after cyclic photocatalytic reaction.**d** XPS spectra of Pd 3d, N 1s, Ti 2p and O 1s for 1%PdN-TiO_2_ (before and after reaction).

**Fig. S18.** The full-scan XPS spectra of TiO_2_, N(30)-TiO_2_, 1%Pd-TiO_2_, and 1%PdN-TiO_2_.

**Fig. S19.** Fitted Pd K3-edge EXAFS spectra of K space for 1%PdN-TiO_2_.

**Fig. S20.** Optimized structural models of TiO_2_, N (30)-TiO_2_, 1%Pd-TiO_2_, and 1%PdN-TiO_2_.

**Fig. S21.** The optimized atomic structural models of TiO_2_, N (30)-TiO_2_, 1%Pd-TiO_2_, and 1%PdN-TiO_2_ photocatalyst with hydrogen adsorption on different active sites.

**Supplementary Table S1**. Fitting results of the Nyquist plots

| **Catalyst** | **Rs** | **Rct** | **CPE-T** | **CPE-P** |
| --- | --- | --- | --- | --- |
| TiO_2_ | 11.41 | 1615300 | 9.9279E-5 | 0.90885 |
| N(30)-TiO_2_ | 17.33 | 739510 | 9.9941E-5 | 0.89652 |
| 1%Pd-TiO_2_ | 20.27 | 409910 | 9.1592E-5 | 0.89471 |
| 1%PdN-TiO_2_ | 11.61 | 115030 | 9.9611E-5 | 0.89897 |

**Supplementary Table S2**. Calculated electronic parameters from Mott-Schottky plots

| **Catalyst** | **E_fb_ (V vs. SCE)** | **N_d_ (cm^-3^)** |
| --- | --- | --- |
| TiO_2_ | -0.84 | 6.06×10^19^ |
| N(30)-TiO_2_ | -0.72 | 9.10×10^19^ |
| 1%Pd-TiO_2_ | -0.46 | 4.67×10^19^ |
| 1%PdN-TiO_2_ | -0.45 | 1.11×10^20^ |

**Supplementary Table S3**. The lifetime of TiO_2_, N-TiO_2_, 1%Pd-TiO_2_ and 1%PdN-TiO_2_ in time-resolved PL test.

| **Catalyst** | **A_1_ (ns)** | **τ_1_** | **A_1_ (ns)** | **τ_2_** | **τ_av_ (ns)** |
| --- | --- | --- | --- | --- | --- |
| TiO_2_ | 57.1149 | 1.2338 | 0.2831 | 25.9745 | 3.57 |
| N(30)-TiO_2_ | 25.1706 | 1.5257 | 0.2694 | 23.3660 | 4.60 |
| 1%Pd-TiO_2_ | 120.8743 | 1.1491 | 0.1278 | 34.8351 | 2.20 |
| 1%PdN-TiO_2_ | 427.0937 | 0.8969 | 0.1734 | 23.1131 | 1.13 |

**Supplementary Table 4.** A comparison of the photocatalytic hydrogen production performance with previously reported Pd-modified-photocatalysts and M-TiO_2_-based (M stands for metal) photocatalysts.

| **Photocatalysts** | **Light source** | **Sacrificial agents** | **PHE rates (mmol g^-1^h^-1^)** | **AQE(%)** | **Ref.** |
| --- | --- | --- | --- | --- | --- |
| Pd_75_Cu_25_/TiO_2_ | 365nm LED lamp | Methanol | 0.269 | 25.77 | [7] |
| Ni cluster -TiO_2_ | Xe lamp (350W) | 20% Methanol | 8.72 | / | [8] |
| 0.5%Pt atom-TiO_2_ | Xe lamp (300W) | 10% Methanol | 9.00 | / | [9] |
| Rh-Cu/TiO_2_ | 254 nm LED lamp | 20% Methanol | 9.26 | / | [10] |
| Pt SA/Def-s-TiO_2_ | 300 W Xe-lamp | 20% Methanol | 13.46 | / | [11] |
| CuSAs-N/TiO_2_ | 300 W Xe-lamp | 40% Methanol | 18.53 | 8.95 | [12] |
| Pt/TiO_2_ | 300 W Xe lamp | 10% Methanol | 22.68 | 57.4 | [13] |
| Pt/TiO_2_-5CTAC | 300 W Xe lamp | 20% Methanol | 24.3 | 3.9 | [14] |
| Co-O-Pt/TiO_2_ | 300 W Xe lamp | 10% Methanol | 43.47 | 83.4 | [13] |
| 0.99%Pt atom-TiO_2_ | 300 W Xenon lamp | 10% Methanol | 52.72 | / | [15] |
| PtAu/{001}-TiO_2_ | 300 W Xenon lamp | 10% Methanol | 61.5 | 72.9 | [16] |
| CuSA-TiO_2_ | 300 W Xenon lamp | 67% Methanol | 101.7 | 56 | [1] |
| Pt/2D TiO_2_ | 300 W Xenon lamp | 20% Methanol | 106.5 | 66 | [17] |
| Pt-Pd SAs/V_o_-TiO_2_ | 300 W Xenon lamp | 10% Methanol | 4.24 | / | [18] |
| PdH_x_/SrTiO_3_ | 300 W Xenon lamp | / | 5 | 10.76 | [19] |
| Pd@TiO_2_@ZnIn_2_S_4_ | 300 W Xenon lamp | / | 5.35 | / | [20] |
| Pd/TiO_2_-V | 300 W Xenon lamp | / | 7.6 | / | [21] |
| Pd@TiO_2_@Au | 300 W Xe lamp | 20% Methanol | 27.23 | / | [22] |
| Pd_0.75_/TiO_2_ | 300 W Xenon lamp | 25% Methanol | 53.64 | 40.16 | [23] |
| PdCu/TiO_2_ | 365 nm LED lamp | 67% Methanol | 94.35 | 83.81 | [24] |
| 1%PdN-TiO_2_ | 365 nm LED lamp | 67% Methanol | 185.8 | 67.4 | This  work |

**Supplementary Table S5**. Fitting parameters of Fourier-transformed Pd L3-edge EXAFS spectra at the for 1%PdN-TiO_2_.

| **Sample** | **Path** | **CN** | **R(Å)** | **σ^2^** | **ΔE_0_** | **R factor** |
| --- | --- | --- | --- | --- | --- | --- |
| 1%PdN-TiO_2_ | Pd-O | 2.5 | 2.03 | 0.004 | -5.5 | 0.018 |
|  | Pd-N | 1.2 | 2.08 | 0.005 | -5.5 |  |

**Supplementary References**

[1]	Y. Zhang, J. Zhao, H. Wang, B. Xiao, W. Zhang, X. Zhao, T. Lv, M. Thangamuthu, J. Zhang, Y. Guo, J. Ma, L. Lin, J. Tang, R. Huang, Q. Liu, *Nature Communications* **2022**, *13* (1), 58.

[2]	Kresse, G.; Joubert, D. From ultrasoft pseudopotentials to the projector augmented- wave method. Physical Review B 1999, 59, 1758-177.

[3]	Perdew, J. P.; Burke, K.; Ernzerhof, M. Generalized gradient approximation made simple. Physical Review Letters 1996, 77, 3865-3868.

[4]	S. Grimme, J. Antony, S. Ehrlich, H. Krieg, *The Journal of Chemical Physics* **2010**, *132* (15), 154104.

[5]	Y. Fan, X. Hao, N. Yi, Z. Jin, *Applied Catalysis B: Environment and Energy* **2024**, *357*, 124313.

[6]	W. H. Lee, C. W. Lee, G. D. Cha, B.-H. Lee, J. H. Jeong, H. Park, J. Heo, M. S. Bootharaju, S.-H. Sunwoo, J. H. Kim, K. H. Ahn, D.-H. Kim, T. Hyeon, *Nature Nanotechnology* **2023**, *18* (7), 754.

[7]	J. Xu, D. Gao, H. Yu, P. Wang, B. Zhu, L. Wang, J. Fan, *Chinese Journal of Catalysis* **2022**, *43* (2), 215.

[8]	J. Si, L. Yu, Y. Wang, Z. Huang, K. Homewood, Y. Gao, *Applied Surface Science* **2020**, *511*, 145577.

[9]	G. Jeantelot, M. Qureshi, M. Harb, S. Ould-Chikh, D. H. Anjum, E. Abou-Hamad, A. Aguilar-Tapia, J.-L. Hazemann, K. Takanabe, J.-M. Basset, *Physical Chemistry Chemical Physics* **2019**, *21* (44), 24429.

[10]	R. Camposeco, M. Hinojosa-Reyes, R. Zanella, *International Journal of Hydrogen Energy* **2021**, *46* (51), 26074.

[11]	X. Hu, J. Song, J. Luo, H. Zhang, Z. Sun, C. Li, S. Zheng, Q. Liu, *Journal of Energy Chemistry* **2021**, *62*, 1.

[12]	B. Xiao, C. Shen, Z. Luo, D. Li, B. Zi, T. Zhou, H. Sun, M. Chen, J. Zhang, Z. Zhu, F. Liu, H. Cui, Q. Liu, *Chemical Engineering Journal* **2024**, *499*, 156331.

[13]	C. Wang, K. Wang, Y. Feng, C. Li, X. Zhou, L. Gan, Y. Feng, H. Zhou, B. Zhang, X. Qu, H. Li, J. Li, A. Li, Y. Sun, S. Zhang, G. Yang, Y. Guo, S. Yang, T. Zhou, F. Dong, K. Zheng, L. Wang, J. Huang, Z. Zhang, X. Han, *Advanced Materials* **2021**, *33* (13), 2003327.

[14]	X. Shao, F. Xiao, X. Zhao, Z. Hou, F. Yue, L. Wang, R. Wu, J. Wang, X. Su, C. Yang, *Nanoscale* **2021**, *13* (2), 901.

[15]	Y. Chen, S. Ji, W. Sun, Y. Lei, Q. Wang, A. Li, W. Chen, G. Zhou, Z. Zhang, Y. Wang, L. Zheng, Q. Zhang, L. Gu, X. Han, D. Wang, Y. Li, *Angewandte Chemie International Edition* **2019**, *59* (3), 1295.

[16]	T. Wei, P. Ding, T. Wang, L.-M. Liu, X. An, X. Yu, *ACS Catalysis* **2021**, *11* (23), 14669.

[17]	G. Zhu, T. Wang, S. Zhang, X. Yang, X. Wang, Y. Zeng, H. Yu, *ACS Applied Materials & Interfaces* **2025**, 17 (42), 58167–58177.

[18]	Y. Zhou, H. Qin, S. Fang, Y. Wang, J. Li, G. Mele, C. Wang, *Catalysis Science & Technology* **2022**, *12* (23), 7151.

[19]	P. C. Ding, H. X. Yang, W. B. Li, Y. Zhang, H. Y. Lin, M. M. Wang, Y. Y. Tang, W. J. Li, H. Y. Yuan, X. L. Wang, S. Dai, P. F. Liu, H. G. Yang, *Nano Letters* **2025**, *25* (16), 6743.

[20]	P. She, J. s. Qin, J. Sheng, Y. Qi, H. Rui, W. Zhang, X. Ge, G. Lu, X. Song, H. Rao, *Small* **2022**, *18* (10), 2105114.

[21]	Z. S. Huang, Y. F. Wang, M. Y. Qi, M. Conte, Z. R. Tang, Y. J. Xu, *Angewandte Chemie International Edition* **2024**, *63* (47), e202412707.

[22]	P. She, J.-s. Qin, H. Rao, B. Guan, J. Yu, *Materials Chemistry Frontiers* **2020**, *4* (6), 1671.

[23]	T. Lv, B. Xiao, F. Xia, M. Chen, J. Zhao, Y. Ma, J. Wu, J. Zhang, Y. Zhang, Q. Liu, *Chemical Engineering Journal* **2022**, *450*, 137873.

[24]	T. Zhou, T. Lv, B. Xiao, L. Duan, Q. Lu, B. Zi, H. Zheng, M. Chen, J. Zhang, H. Sun, Y. Zhang, L. Zhang, T. He, G. Hu, Q. Liu, *Chemical Engineering Journal* **2023**, *478*, 147372.
